# Supplementary material for: Influence of chirality and sequence in lysine-rich lipopeptide biosurfactants and micellar model colloid systems
Source: Nat Commun. 2024 Aug 8;15:6785. doi: 10.1038/s41467-024-51234-8 (PMC11310517; doi:10.1038/s41467-024-51234-8)
Supplement: Supplementary file 1 — Supplementary Information [file 41467_2024_51234_MOESM1_ESM.docx]

**Supplementary Information**

**Influence of Chirality and Sequence in Lysine-Rich Lipopeptide Biosurfactants and Micellar Model Colloid Systems**

Ian W Hamley^1,*^ Anindaysundar Adak,^1^ Valeria Castelletto,^1^

*^1^ School of Chemistry, Food Biosciences and Pharmacy, University of Reading, Whiteknights, Reading RG6 6AD, U.K.*

**Supplementary Fig. 1. Scaled SAXS data.** SAXS data for **P2D** with intensity scaled by concentration.

**Supplementary Fig. 2**. **Structure factor analysis from SAXS data**. Comparison of fits to SAXS data for 5 wt% **P2D** using hard-sphere and Hayter-Penfold structure factors. (a) Data with fits, (b) Residuals. For ease of visualization, only every 5^th^ data point is shown.

**Supplementary Fig. 3**. **Concentration-dependent zeta potential**. Measured zeta potential values as a function of concentration.

**Supplementary Fig. 4**. **Comparison of measured SAXS data with that computed from MD configurations.** Comparison of measured data for **P1** and **P2** 0.2 wt% (thin lines) with calculated form factor using CRYSOL based on MD simulation configurations with *p* = 70 for the two molecules. The data for **P2** are shifted by division by a factor of 10. A flat background term BG = 10^-4^ was added to the CRYSOL calculated data in both cases.

**Supplementary Fig. 5**. **SASA and solvation-related properties calculated from MD simulations**. Solvent-accessible surface area (SASA), Gibbs energy change of solvation ΔG(solv), volume and density for (a) **P1**, (b) **P1D**, (c) **P2**, (d) **P2D**.

**
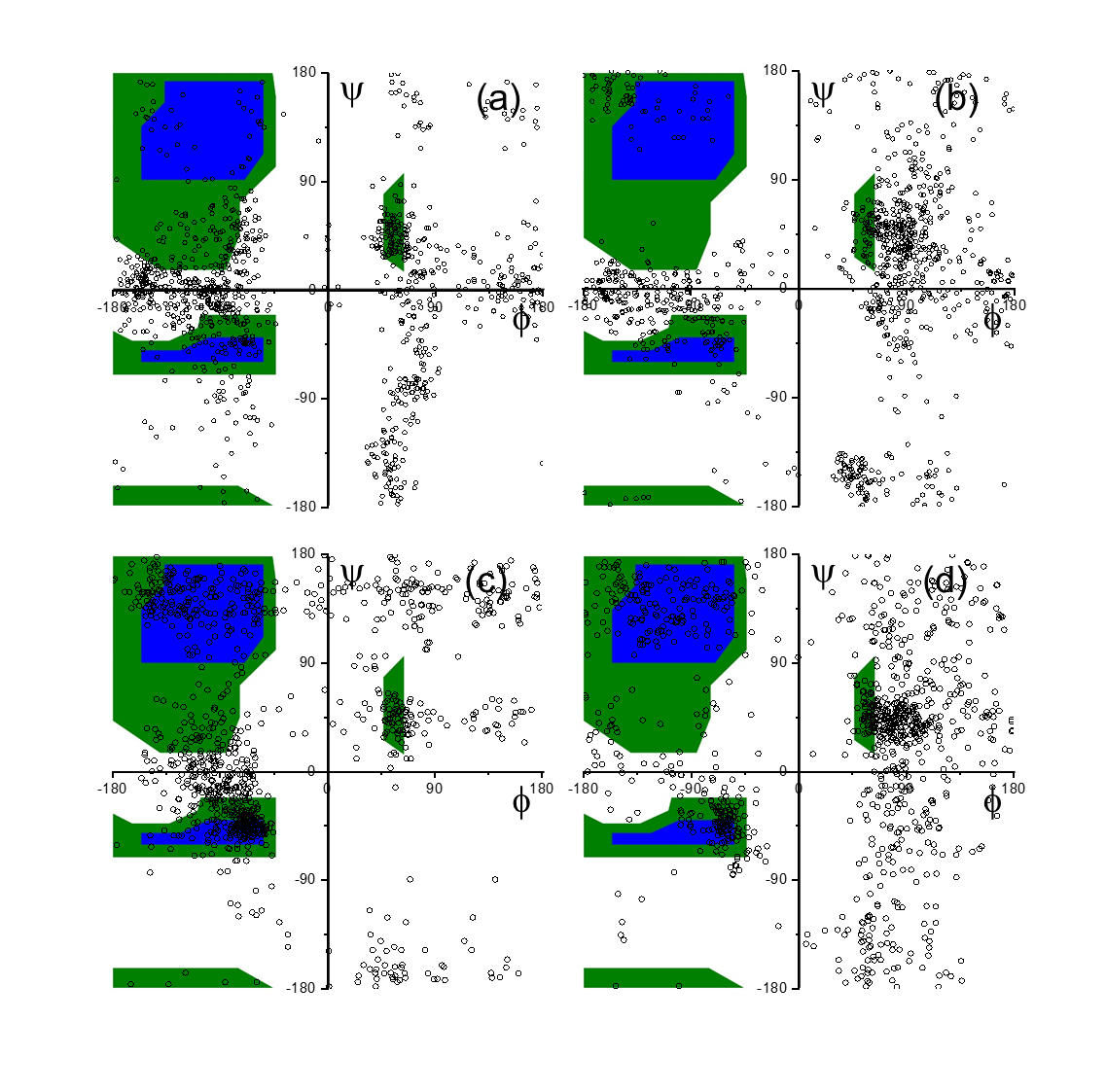
**

**Supplementary Fig. 6. Ramachandran plots for aromatic residues**. Dihedral angles from simulation frames from *t* = 900 ps to *t* = 1000 ps) computed from MD simulations. (a) **P1** Tyr (b) **P1D** Tyr, (c) **P2** Trp, (d) **P2D** Trp.

**Supplementary Table 1**. **Least-squares fit parameters for SAXS data.** For 5 wt% **P2D** using hard-sphere and Hayter-Penfold structure factors.

| Model    Parameters | 5% **P2D**  Hayter-Penfold | 5% **P2D**  Hard sphere |
| --- | --- | --- |
| *R*_o_ ± σ / Å | 29.61±3.11 | 31.88±4.08 |
| *R*_i_ / Å | 15.06 | 10.45 |
| *μ* | -1.480 | -4.606 |
| *η* / cm^-1^ | 9.516×10^-6^ | 8.501×10^-6^ |
| BG | 4.279×10^-3^ | 1.000×10^-3^ |
| *R*_HS_ / Å | 45.41 | 55.78 |
| *φ* | 0.1616 | 0.7000 |
| *z*_eff_ | 63.50 | N/A |
| *I* | 0.1000 | N/A |

**Key:** **Form factor**: *R*_o_*:* outer radius (*σ*_c_ Gaussian polydispersity in *c*), *R*_i_: inner radius, *μ*: ratio of scattering contrast of inner core/outer core, *η*: scattering contrast of core, BG: constant background. **Structure factor**: *R*_HS_, hard sphere radius, *φ*: effective volume fraction *z*_eff_: effective charge, *I*: ionic strength (temperature fixed at *T* = 293 K).
